# Sex-dependent effects of Canagliflozin on kidney protection in mice with combined hypertension-type 1 diabetes

**DOI:** 10.1371/journal.pone.0295284

**Published:** 2023-12-06

**Authors:** Mayra Trentin-Sonoda, Véronique Cheff, Alex Gutsol, Richard L. Hébert

**Affiliations:** 1 Kidney Research Centre, Division of Nephrology, Department of Medicine, Ottawa Hospital Research Institute, Ottawa, Ontario, Canada; 2 Department of Cellular and Molecular Medicine, University of Ottawa, Ottawa, Ontario, Canada; Max Delbruck Centrum fur Molekulare Medizin Berlin Buch, GERMANY

## Abstract

Canagliflozin (CANA) is a sodium-glucose cotransporter 2 (SGLT2) inhibitor with blood glucose lowering effects. CANA also promotes kidney protection in patients with cardiovascular diseases and type 2 diabetes (T2D), as well as in normoglycemic patients with hypertension or heart failure. Clinical studies, although conduct in both sexes, do not report sex-dependent differences in T2DM treated with CANA. However, the impact of CANA in type 1 diabetes, as well in sex-dependent outcomes in such cohort needs further understanding. To analyze the effects of CANA in mice with combined hypertension and type 1 diabetes, diabetes was induced by STZ injection (5 days, 50mg/kg/day) in both male and female 8 weeks old genetic hypertensive mice (Lin), whereas the control (Lin) received 0.1M sodium citrate injections. 8 weeks after STZ. Mice were fed either regular or CANA-infused diet for 4 weeks. 8 weeks after STZ, hyperglycemia was present in both male and female mice. CANA reversed BG increase mice fed regular diet. Male LinSTZ mice had elevated water intake, urine output, urinary albumin to creatinine ratio (ACR), kidney lesion score, and creatinine clearance compared to the Lin control group. Kidney injury was improved in male LinSTZ + CANA group in male mice. Water intake and urine output were not statistically significantly different in female LinSTZ compared to female LinSTZ+ CANA. Moreover, CANA did not improve kidney injury in female mice, showing no effect in creatinine clearance, lesion score and fibrosis when compared to LinSTZ fed regular diet. Here we show that Canagliflozin might exert different kidney protection effects in male compared to female mice with hypertension and type 1 diabetes. Sex-dimorphisms were previously found in the pathophysiology of diabetes induced by STZ. Therefore, we highlight the importance of in-depth investigation on sex-dependent effects of CANA, taking in consideration the unique characteristics of disease progression for each sex.

## Introduction

The worldwide prevalence of diabetes mellitus (DM) is a major concern in the modern society [1], and one of the leading causes of chronic kidney disease (CKD). Diabetic kidney disease (DKD) is a multifactorial disease. DKD is accompanied by glomerular hyperfiltration, increased intraglomerular pressure, and osmotic imbalance. Hypertension is closely related with the development of chronic kidney disease and to end stage renal disease (ESRD) [2, 3]. Together, diabetes and hypertension increase the mortality rate associated to cardiovascular complications [4].

In normoglycemic kidneys, the sodium glucose co-transporter 2 (SGLT2), present in the S1, S2 segments of proximal tubular cells, is responsible for 90–95% of glucose reabsorption [5, 6]. In hyperglycemic kidneys, blood glucose levels are elevated and SGLT2 is overexpressed as a compensatory mechanism [7, 8]. Making SGLT2 inhibitors an excellent therapeutic candidate for patients with diabetes type 2 (T2D). Moreover, SGLT2 inhibitors has shown to promote nephroprotection on normoglycemic patients, indicating its overall therapeutic potential against kidney diseases ([9, 10]).

Gliflozins are a class of drugs with SGLT2 inhibitory properties primarily applied for glycemic control [11], with beneficial effects in patients with cardiovascular diseases and T2D, as well as cardiovascular complications [12, 13]. Dapagliflozin has shown to be protective against end-stage kidney disease, or even death against renal or cardiovascular diseases in patients with or without T2D [10]. Clinical studies do not report sex-dependent differences in T2DM treated with CANA [14, 15]. Furthermore, a recent systematic review (SR) has highlighted the lack of studies involving SGLT2 inhibitors in females rodents, as well as emphasized the importance of understanding the sensitivity of that class of drugs in both male and female animals [16]. Thus, stressing the need for further understanding the impact of CANA in type 1 diabetes and/or sex-dependent outcomes in such cohort.

Here we aimed to study the effects of CANA on kidney function and structure in a model of combined hypertension-type 1 diabetes (LinSTZ). In this study of exploratory character, we hypothesized that CANA would decrease blood glucose, thus improving electrolytes, ameliorating glomerular hyperfiltration, improving albuminuria and kidney injury compared to the LinSTZ group independent of sex. We report that CANA lowers blood glucose levels in both sexes. However, CANA was not able to normalize hyperfiltration in female mice. CANA exerted kidney protective effects in male LinSTZ mice, but not in females. We hypothesize that time of intervention, as well as sex-dimorphisms in the pathophysiology of hypertension-diabetes are key to explain the findings of this study. Further investigation is necessary to understand the mechanisms by which CANA protects against hyperfiltration and kidney injury, hence, improving kidney function and repair mechanisms.

## Methods

### *In vivo* study

In this study, we used a mice model of genetic hypertension (TTRhRen or Lin) generated by Prescott and colleagues [17], previously characterized in the literature [17, 18]. Briefly, the transgene construct consists of a modified human pro-renin cDNA transgene, under the control of a 3-Kb region of the mouse liver-specific transthyretin promoter.

Lin mice were bred and housed in the University of Ottawa animal facility. In total, 39 mice (male and female). Eight to ten weeks-old Lin on a FVB/n background were randomly allocated to groups Lin (3 males, 4 females), LinSTZ (12 males, 4 females), and LinSTZ + CANA (12 males, 4 females). Lin mice were subjected to 5 days of intraperitoneal (i.p.) injections of STZ (50mg per kg of body weight; cat. S0130, Sigma-Aldrich, Oakville, ON.) to induce hyperglycemia via pancreatic beta cell death [19–21]. Control Lin mice received 0.1 M Na-citrate buffer pH 4.5 instead of STZ. 8 weeks post-STZ injections, Lin and LinSTZ mice fed either a regular diet (10% kilocaloric (kCal); Teklad, Mississauga, ON) and LinSTZ + CANA mice were fed Canagliflozin (cat. A8333, APExBIO)-infused diet (225ppm, Envigo, Indianapolis, IN, United States) for 4 weeks. All mice were subjected to endpoint metabolic cages where food consumption, water intake and urine output data were collected at 24 hours. To prevent distress, mice were monitored throughout the study and were euthanized before they reached the humane endpoint. At the first display of dehydration in the hypertension/ hyperglycemic groups, mice were given with i.p. saline solution and accommodated on cages with heat pads for up to 8 hours at the time. Out of the 32 mice treated with STZ, 1 male in the group LinSTZ and 4 males in the group LinSTZ + CANA showed severe signs of dehydration and reached humane endpoint 7–9 weeks post STZ.

Anesthesia was induced with 5% isoflurane and euthanasia occurred by exsanguination (cardiac puncture); blood and tissue were collected and further allocated for different assays. Kidneys were collected in 4% PFA for histological analysis or snap frozen in liquid nitrogen for future analysis.

Experimental animals were bred and housed at the Animal Care Facility at the University of Ottawa (under protocol number CMM-3810) with free access to food and water. Ethical approval was obtained from the University of Ottawa Animal Care Committee (under protocol number CMM-3809) and the study was conducted according to the guidelines of the Canadian Council on Animal Care.

### Blood pressure measurement

Systolic blood pressure was measured by tail-cuff plethysmography (RRID:SCR_022985, BP 2000, Visitech systems, Apex, NC) [22]. Mice were trained for 5 consecutive days 5 preliminary readings, 10 actual readings/day). Later BP measurement in the study was obtained within 2 consecutive days on 12- and 20-weeks old mice. False results from unread blood pressure instrument and outliers measured according to the average ± two-times standard deviation of sample were removed from data analysis to obtained sample average. Only one female mouse from LinSTZ + CANA group did not display readings at endpoint.

### Biochemistry analysis

Blood samples were collected at endpoint via cardiac puncture into heparinized syringes, kept on ice and centrifuged at 5000 g for 10 minutes at 4°C. The plasma fraction was immediately frozen at -80°C until subsequent analysis. Plasma (cholesterol, creatinine, glucose, potassium, triglycerides, AST, ALT) and urine (electrolytes, urine urea nitrogen, creatinine, glucose) biochemistry tests were performed by IDEXX inc. (IDEXX, Westbrook, Maine). The values of the urine obtained for biochemistry were normalized for the 24 hours urine output (obtained by metabolic cages, as described before).

### Albuminuria

Albuminuria was measured using the Mouse Albumin Elisa Kit (cat. E99-134, Bethyl labs, Montgomery, TX.) following manufacturer’s protocol. Albuminuria was determined by calculated urine Albumin to creatine ratio.

### eGFR

Glomerular filtration rate was estimated by FITC-inulin method, following the protocol from the Animal Models of Diabetic Complications Consortium. Briefly, mice were injected FITC-inulin (cat. F3272, Sigma-Aldrich, Oakville, ON, Canada) intravenously, followed by saphenous bleeds at 3, 7, 10, 15, 25, 55, and 75 minutes post-injection. GFR was calculated based on inulin counts throughout bleeds, according to the literature [23].

### Injury score and fibrosis

At endpoint, kidneys were removed, individually weighted, and normalized to tibia length. Tibias were collected at endpoint (after euthanasia occurred) and measured with the aid of a digital caliper). Kidney weight was then normalized by tibia length, expressed in g/mm. A section of the right kidney was stored in 4% paraformaldehyde fixation solution for 24 hours, followed by another 24h in 70% ethanol before embedded in paraffin. Staining with periodic-acid Schiff (PAS) or Masson’s Trichrome assays was performed in PFA Paraffin-embedded kidney sections (3 μm). All sectioning, paraffin embedding, and staining were performed by the University of Ottawa’s Department of Pathology. The sections were visualized using a light microscope at either 200X (Axioskop 2 Imager A1, Zeiss, Germany. Injury score was classified based on degree of kidney damage, using the following qualitative scale: 0—no damage in tissue, 1—minimal, 2—mild, 3—moderated, 4—marked and 5—severed damage and injury. Two midhilar coronal cross sections of each kidney (i.e. from each animal) were used to collect data from 20–30 cortical nonconfluent view fields (i.e. 120–150 measurements per group). Representative areas were analyzed in a blinded manner.

### Statistics

GraphPad Prism (Version 9.3.1, Graphpad Prism, San Diego, Ca) was used to present the data and perform statistical analysis. Values within the text are presented as mean difference and 95% CI of difference Values in the figures are expressed as means ± standard deviation (SD). One-way or ANOVA test was applied for single time point, followed by Tukey post-test. Due missing values from mortality throughout the study, multiple timepoints were analyzed by mixed-effects analysis, followed by Fisher’s LSD test. A P-value < 0.05 was considered statistically significant.

## Results

### Canagliflozin lowers blood glucose levels but has no effect on systolic blood pressure of LinSTZ mice

**Fig 1:**
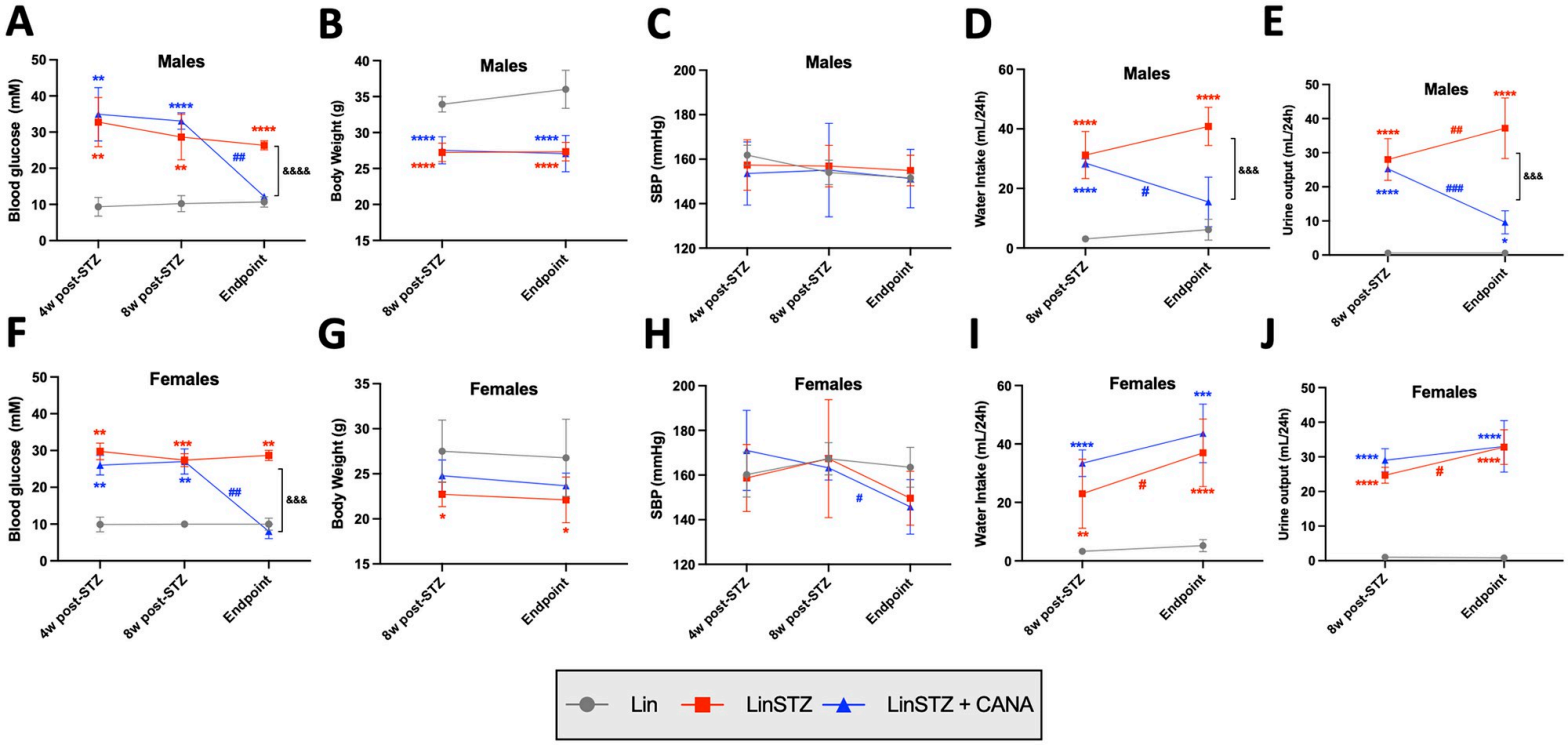
Effects of CANA in LinSTZ males A) blood glucose, B) body weight, C) Systolic blood pressure (SBP), D) water intake, and E) 24h urine output, as well as in females F) blood glucose, G) body weight, H) SBP, I) water intake, and J) 24h urine output at timepoints 4 weeks and/or 8 weeks after STZ or 0.1M sodium citrate injections, as well as endpoint Data presented as Mean ± SD. **P<0.01, ***P<0.001, ****P<0.0001 vs respective timepoint Lin group; # P<0.05, ## P<0.01, and ###P<0.001 vs respective 8w post-STZ; &&& P<0.001 and &&&&P<0.0001 vs endpoint LinSTZ. N 3 to 10 per group.

We first confirmed that STZ was able to induce hyperglycemia by monitoring blood glucose (BG) levels 4 and 8 weeks after STZ injections. BG was increased in both male and female LinSTZ and LinSTZ+CANA groups in both time-points (Fig 1A and 1F). CANA reversed hyperglycemic state in both male and female LinSTZ mice [Endpoint vs 8w post-STZ, male: -20.9 (-24.5 to -17.3, P<0.01) and females -19.0 (-24.3 to-13.78, P<0.01) (Fig 1A and 1E). Male STZ groups had decreased body weight compared to Lin control (Fig 1B), whereas CANA treatment did not affect body weight (Fig 1B and 1G). Blood pressure was also monitored, but no changes were found in males (Fig 1C). Endpoint vs 8w post-STZ mean difference on SBP of female LinSTZ was -17.7 (-94.4 to 59.0, ns) and LinSTZ + CANA was -17.5 (-29.2 to -5.8, P<0.05 (Fig 1G). However, no differences were found between LinSTZ and LinSTZ + CANA at endpoint [-3.9 (-28.7 to 20.8)].

We have previously shown that LinSTZ mice have increased water intake and urine output [24]. To investigate whether CANA would have an effect in polydipsia and/or polyuria, mice were placed in metabolic cages for 24h, prior to the endpoint. Male and Females STZ mice had increased water consumption and urine output compared to the control Lin (Fig 1D and 1I); CANA reversed polydipsia in male, but not female LinSTZ [Fig 1D and 1I; endpoint vs 8w post-STZ, male: -13.0 (-23.0 to -3.0, p<0.05) and female 10.2 (-2.0 to 22.3, p = 0.0946)]. A similar pattern was observed with urine volume, where female and male LinSTZ displayed polyuria, while male mice treated with CANA displayed lower urine volume compared to LinSTZ [Fig 1E and 1J; endpoint vs 8w post-STZ, male -15.5 (-21.5 to—9.5, p<0.001), females 4.0 (-2.6 to 10.6)].

### Effects of CANA in glomerular filtration rate and electrolytes

Both hypertension and hyperglycemia induce changes in kidney hemodynamics. To assess the effects of CANA in kidney function, we first evaluated glomerular filtration and urine biochemistry.

LinSTZ mice fed regular diet did not show changes eGFR compared to Lin control (Table 1), whereas CANA increased eGFR in females (Table 1). Creatinine clearance (CrCl) was increased in both male and female LinSTZ mice (Table 1). Male LinSTZ + CANA mice had lower levels of CrCl than LinSTZ (Table 1).

**Table 1:** Glomerular filtration, urinary glucose and electrolytes at endpoint.

	LinSTZ vs Lin				LinSTZ + CANA vs Lin				LinSTZ + CANA vs LinSTZ			
	Males		Females		Males		Females		Males		Females	
	Mean dif.	95% CI of dif.	Mean dif.	95% CI of dif.	Mean dif.	95% CI of dif.	Mean dif.	95% CI of dif.	Mean dif.	95% CI of dif.	Mean dif.	95% CI of dif.
*eGFR (ml/min/gBW)*	10.8	-5.3 to 26.8	7.4	-1.0 to 15.8	3.7	-13.4 to 20.9	8.5*	0.1 to 16.9	-7.0	- 23.1 to 9.0	1.1	- 6.7 to 8.9
*Creatinine clearance (ml/min)*	7.2**	2.4 to 12.1	6.3*	0.8 to 11.9	2.4	-3.0 to 7.7	8.9**	3.4 to 14.5	-4.9*	-9.0 to -0.8	2.6	- 2.5 to 7.7
*Urine osmolarity (mOsm/Kg)*	-886.3 ***	-1224.0 to -548.5	-427.0	-1696.0 to 842.2	-735.3 **	-1097.0 to -374.1	-483.5	-1671.0 to 703.7	151.0	-186.9 to 488.9	-56.5	-1244.0 to 1131.0
*Creatinine (mg/dL)*	-47.1**	-69.6 to -24.6	-19.75	-40.4 to 0.9	-43.0**	-67.1 to -18.9	-25.8*	-46.4 to -5.1	4.1	-18.4 to 26.6	-6	- 26.6 to 14.6
*Glucose/Cr (mg/mg)*	731.3 ***	273.0 to 1190.0	752.7 ***	431.5 to 1074.0	376.6	-113.3 to 886.5	735.1 ***	437.7 to 1033.0	-354.6	- 812.9 to 103.6	-17.6	- 338.8 to 303.7
*Calcium/Cr (mEq/mg)*	0.007	-0.007 to 0.021	0.013	-0.014 to 0.040	0.006	-0.009 to 0.022	-0.011	-0.038 to 0.016	-0.001	-0.015 to 0.014	-0.024	-0.051 to 0.003
*Chloride/Cr (mEq/mg)*	0.51*	0.08 to 0.93	0.05**	0.01 to 0.08	0.04	-0.41 to 0.49	0.02	-0.1 to 0.06	-0.47	-0.89 to -0.05	-0.02	-0.05 to 0.01
*Magnesium/Cr (mEq/mg)*	0.08**	0.05 to 0.12	0.09**	-0.03 to 0.15	0.08**	0.05 to 0.12	0.02	-0.04 to 0.07	-0.01	-0.04 to 0.03	-0.07*	-0.13 to -0.02
*Phosphorus/Cr (mEq/mg)*	0.42**	0.23 to 0.62	0.47	-0.04 to 0.97	0.23*	0.10 to 0.56	0.49	-0.13 to 1.00	-0.09	-0.31 to 0.13	0.03	-0.48 to 0.54
*Potassium/Cr (mEq/mg)*	0.25*	0.02 to 0.48	0.30**	0.11 to 0.48	0.03	-0.21 to 0.28	0.26**	0.08 to 0.44	-0.21	-0.44 to 0.02	-0.04	-0.22 to 0.15
*Sodium/Cr (mEq/mg)*	0.33 ***	0.19 to 0.47	0.30	-0.09 to 0.80	0.28**	-0.12 to 0.42	0.09	-0.31 to 0.48	-0.05*	-0.19 to -0.09	-0.22	-0.62 to 0.17
*Urea Nitrogen/Cr (mEq/mg)*	68.2*	10.0 to 126.5	52.4**	18.4 to 86.4	5.5	-56.8 to 67.7	49.5**	-15.5 to 83.5	-62.8*	-121.0 to -4.5	-2.9	-36.9 to 31.1

eGFR: estimated glomerular filtration ratio, Cr: creatinine.

*P<0.05,

** P<0.01,

***P<0.001

To assess urine concentration, we measured urine osmolarity. Decreased urine osmolarity was present in LinSTZ males, while unchanged in female compared to Lin group (Table 1). No changes were observed with CANA treatment compared to Lin or LinSTZ (Table 1). Urine levels of creatinine were decreased in males LinSTZ, LinSTZ + CANA, and females LinSTZ + CANA compared to Lin (Table 1).

In normoglycemia, around 90% of the glucose is reabsorbed in the kidney tubules [5]. In hyperglycemia, excess glucose is excreted in the urine. LinSTZ mice had elevated glucose urine levels in both male and female cohorts (Table 1). Urine glucose levels did not differ between males LinSTZ and LinSTZ + CANA, while remaining elevated in females treated with CANA (Table 1).

Abnormal electrolyte homeostasis is commonly present in diabetes. We evaluated endpoint electrolyte levels in urine as it shows in Table 1. Calcium levels remained unchanged across treatments, independent of sex. Chloride excretion was increased in LinSTZ groups, while it was decreased in males treated with CANA (Table 1). Magnesium levels were higher in both male LinSTZ and male LinSTZ + CANA groups. No changes were observed for females. The same pattern was observed for phosphorus. Female phosphorus levels showed a trend to increase in both LinSTZ and LinSTZ + CANA, compared to Lin (Table 1). Potassium levels were higher in both male and female LinSTZ. Male LinSTZ + CANA had potassium levels similar to the Lin group (Table 1), while females LinSTZ + CANA potassium levels were increased compared to Lin (Table 1). Urinary sodium levels were elevated in males LinSTZ and LinSTZ + CANA. Urea nitrogen in urine was higher in male LinSTZ compared to Lin, while reduced in CANA treated group compared to LinSTZ. In females, Urea nitrogen was elevated in both LinSTZ and LinSTZ + CANA group.

### CANA ameliorates albuminuria and injury score in male LinSTZ

Taking in consideration the kidney protective effect of SGLT2 inhibitors, we further tested our hypothesis by analyzing the effects of CANA on kidney injury and morphometry in hypertensive-diabetic mice. Therefore, we looked at albuminuria (albumin to creatinine ratio—ACR), injury score, fibrosis, and kidney weight.

#### Males

**Fig 2:**
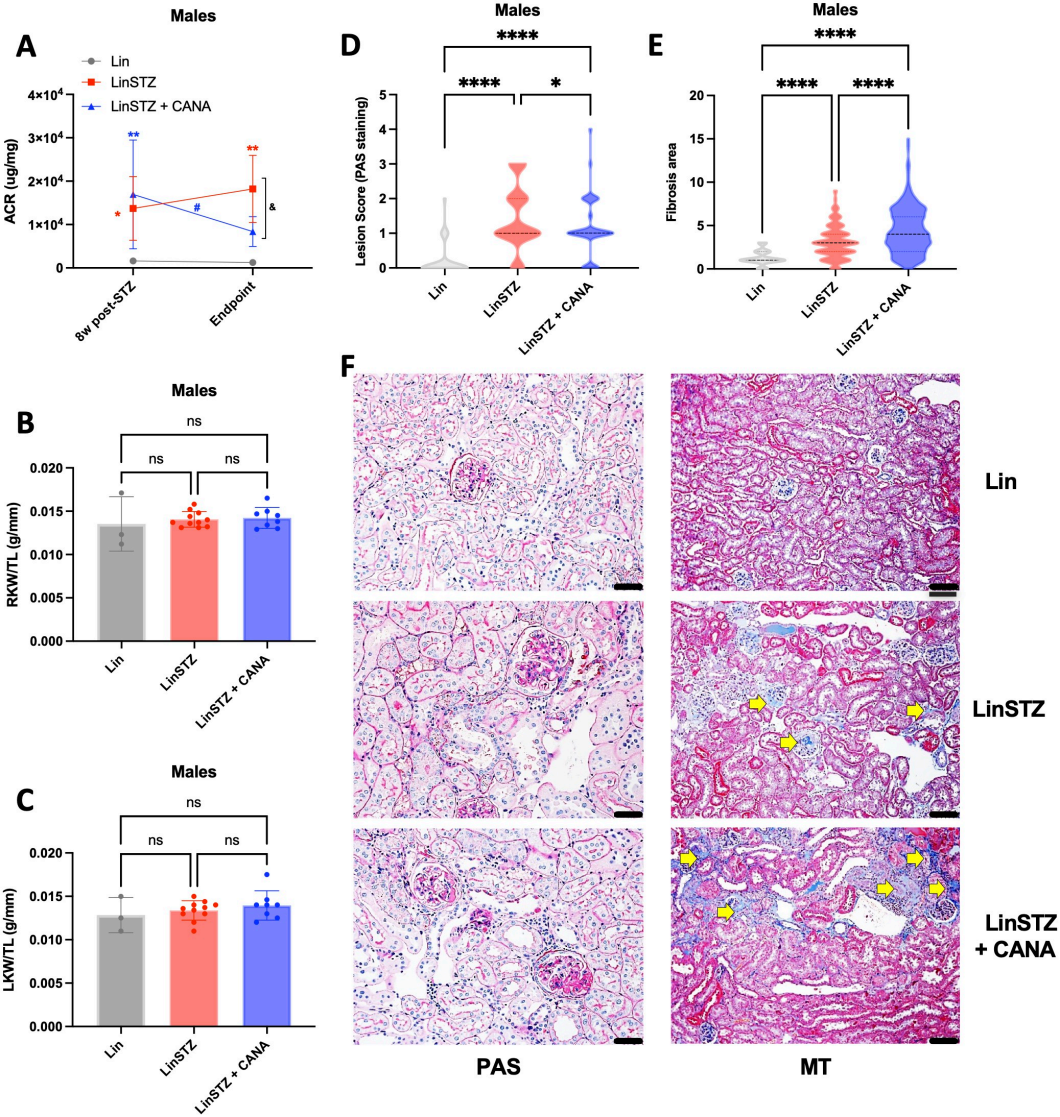
Effects of Canagliflozin in Males A) 8w post-STZ and endpoint albumin to creatinine ratio (ACR), B) endpoint right kidney weight to tibia length ratio, C) endpoint left kidney weight to tibia length ratio, D) endpoint Periodic acid-Schiff (PAS) lesion score, and E) endpoint kidney fibrosis (Mason trichrome staining—MT), and F) representative images of PAS and MT staining, magnification = 200X, scale bar = 50μm. Data presented as Mean ± SD. *P<0.05, **P<0.01, ***P<0.001, ****P<0.0001 vs respective timepoint Lin group; # P<0.05, ## P<0.01, vs respective 8w post-STZ; and &P<0.05 vs endpoint LinSTZ. N 3 to 11 per group. D-E: 20–30 cortical nonconfluent view fields per mouse (3 to 4 mice per group). Yellow arrows indicate fibrotic staining. Within the bar graphs, each dot represents a measurement from one mouse.

ACR was increased in the LinSTZ group at 8 weeks post-STZ injections [LinSTZ vs Lin 12109 (1149 to 23069, p<0.05); LinSTZ + CANA vs Lin: 15336 (4165 to 26508), whereas CANA reversed ACR at endpoint [endpoint vs 8w post-STZ: -7680 (-1436 to -824.5), Fig 2A]. No differences were found in kidney weight to tibia length ratios (Fig 2B and 2C). LinSTZ mice had increased levels of injury score compared to Lin control group (Fig 2D and 2F), whereas CANA exerted partial protection [LinSTZ vs Lin: 1.24 (0.87 to 1.61), p<0.00001; LinSTZ + CANA vs LinSTZ: -0.30 (-0.58 to -0.021, p<0.05)]. LinSTZ and LinSTZ + CANA had the same levels of fibrosis (Fig 2E and 2F).

#### Females

**Fig 3:**
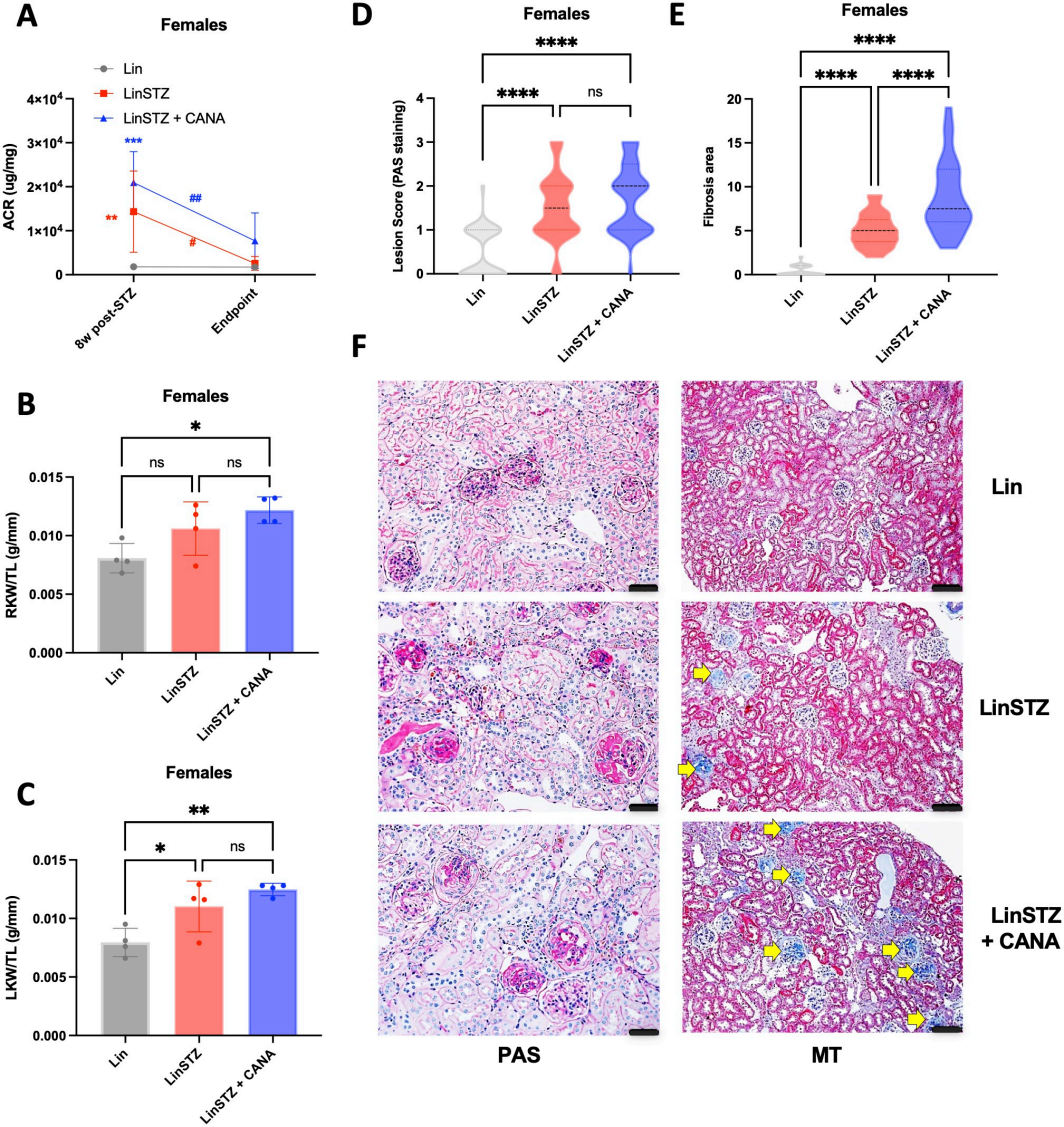
Effects of Canagliflozin in Females A) 8w post-STZ and endpoint albumin to creatinine ratio (ACR), B) endpoint right kidney weight to tibia length ratio, C) endpoint left kidney weight to tibia length ratio, D) endpoint Periodic acid-Schiff (PAS) lesion score, and E) endpoint kidney fibrosis (Mason trichrome staining—MT), and F) representative images of PAS and MT staining, magnification = 200X, scale bar = 50μm. Data presented as Mean ± SD. *P<0.05, **P<0.01, ***P<0.001, ****P<0.0001 vs respective timepoint Lin group; # P<0.05, ## P<0.01, vs respective 8w post-STZ. N = 4 per group (A-C). D-E: 20–30 cortical nonconfluent view fields per mouse (3 to 4 mice per group). Yellow arrows indicate fibrotic staining. Within the bar graphs, each dot represents a measurement from one mouse.

At 8w post-STZ injections, both LinSTZ and LinSTZ+CANA group had elevated ACR levels [LinSTZ vs Lin 12461 (3898 to 21024), P = 0.071; LinSTZ + CANA vs Lin 19110 (11177 to 27043), P = 0.001]. At endpoint, There was no statistically significant differences between LinSTZ or LinSTZ + CANA compared to Lin (Fig 3A). Both LinSTZ and LinSTZ + CANA showed a decrease in ACR from 8w post-STZ to endpoint [LinSTZ: -11746 (-20892 to -2600) P = 0.0189, LinSTZ + CANA: -13236 (-21645 to -4827), P = 0.0074). Right kidney weight to tibia length ratio was increased only in LinSTZ + CANA (Fig 3B), and left kidney weight to tibia length in both LinSTZ and LinSTZ + CANA (Fig 3C). Both LinSTZ and LinSTZ + CANA groups had increased kidney injury score (Fig 3D and 3E). Fibrosis was present in the LinSTZ group and even more pronounced after treatment with CANA (Fig 3D and 3E).

### Canagliflozin in triglycerides and ALT in LinSTZ mice

**Fig 4:**
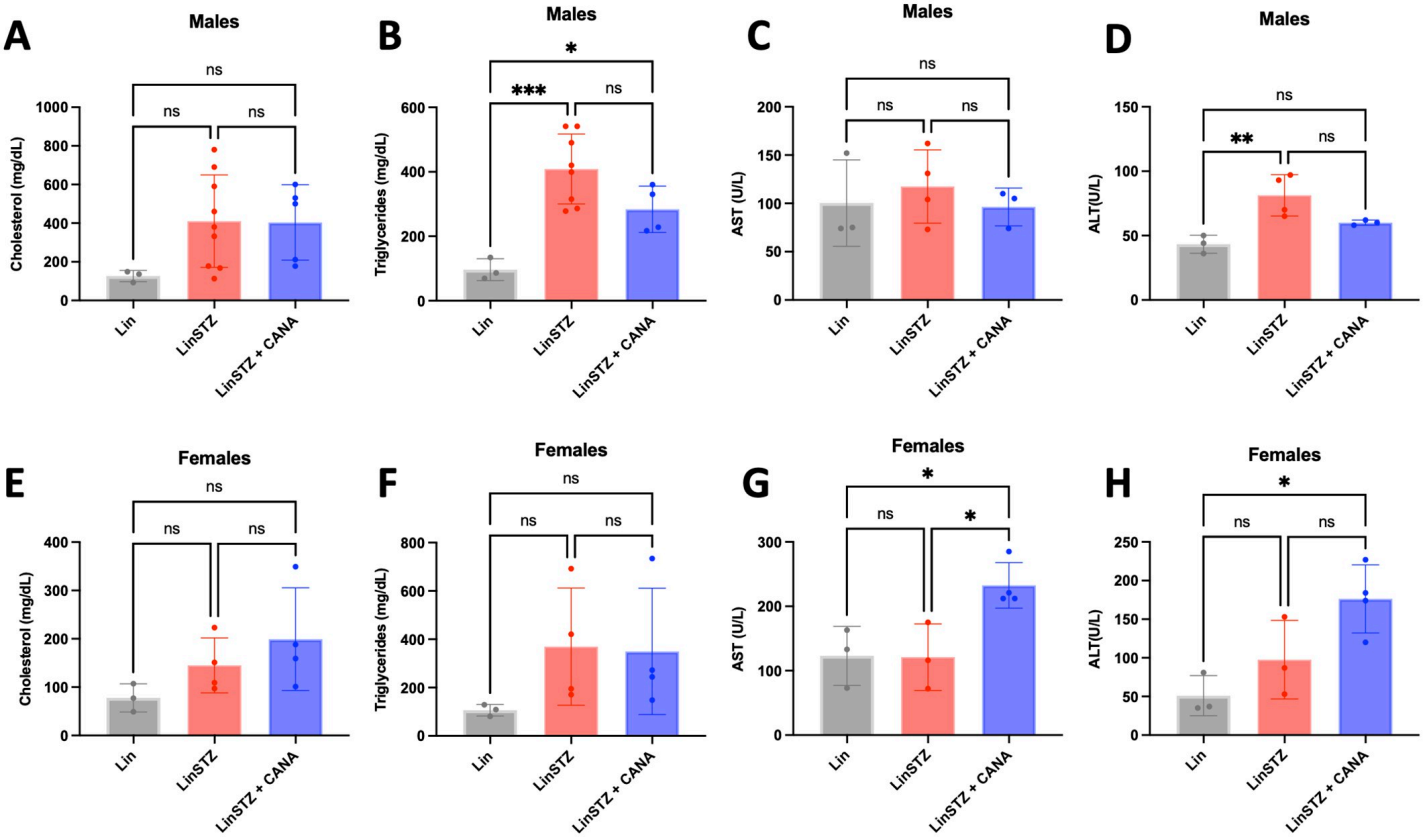
Effects of canagliflozin in endpoint lipidemia and liver enzymes. Serum A) cholesterol, B) triglycerides, C) alanine aminotransferase (AST), and D) alanine transaminase (ALT) in males. Serum E) Cholesterol, F) triglycerides, G) AST, and H) ALT in females. Data presented as Mean ± SD. *P<0.05, **P<0.01, and ***P<0.001. N 3 to 9 per group. Within the bar graphs, each dot represents a measurement from one mouse.

Besides hyperglycemia, impaired insulin production can induce several metabolic alterations. Hyperlipidemia is common in diabetes, representing a risk for cardiovascular events. In males, the mean difference in cholesterol was 284.4 (-80.5 to 648.7) for LinSTZ vs Lin and 277.8 (-121.6 to 677.2) for LinSTZ + CANA vs Lin (Fig 4A). Triglycerides were elevated in the LinSTZ group [312.4 (147.1 to 477.7, P<0.001) for and LinSTZ vs LinSTZ] and showed a trend to decreased in CANA-fed mice [-125.0 (-274.5 to 24.5), with a P = 0.11 for LinSTZ + CANA vs LinSTZ] (Fig 4B). AST levels were unchanged in males, while ALT was increased in male LinSTZ vs Lin [38.0 (12.7 to 63.2), p<0.01] (Fig 4D).

While cholesterol levels in LinSTZ fed-regular diet were similar to Lin group, a trend to increase was observed in mice treated with CANA [LinSTZ vs Lin: 67.3 (-97.8 to 231.4), P = 0.50, LinSTZ + CANA vs Lin 121.6 (-42.5 to 285.7), p = 0.15] (Fig 4B). A trend was also observed for LinSTZ groups regarding triglycerides levels (LinSTZ vs Lin: 263.4 (-214.3 to 741.1), P = 0.31; LinSTZ + CANA vs Lin 244.2 (-233.5 to 721.8), P = 0.35] (Fig 4F). CANA treatment increased AST [LinSTZ + CANA vs Lin 109.5 (11.6 to 207.4), P = 0.0289] and ALT (LinSTZ + CANA vs Lin 78.6 (-15.8 to 173.0), P = 0.0141] in female mice, compared to Lin control (Fig 4G and 4H).

## Discussion

Diabetes is a multifactorial condition, commonly leading to renal dysfunction. When associated with hypertension, it can increase the risk of developing chronic kidney diseases. Although inhibitors of sodium glucose co-transporter were initially utilized for lowering blood glucose levels, clinical studies also found protective effects against cardiovascular events, heart failure, and liver disease [13, 15, 25]. In this study, we aimed to investigate the effects of CANA on kidney function and injury, in a mouse model of combined hypertension and type 1 diabetes. We observed that (1) CANA lowered blood glucose in both female and males LinSTZ; (2) Lower blood glucose levels were accompanied by an improvement of polydipsia and polyuria in males treated with CANA; (3) Electrolytes imbalance was partially restored in mice treated with CANA, with a more pronounced response in males; (4) CANA promoted kidney protection in males, decreasing ACR and kidney injury. And (5) CANA alleviates metabolic changes related to diabetes (triglycerides and AST) in males, but not in females. Altogether, we demonstrate a clear sex-specific response to CANA in the LinSTZ mouse model.

Here we used an established model of type-1 diabetes (T1DM) where streptozocin (STZ) induces cell death of β-pancreatic cells, leading to decreased insulin production, thus promoting elevated blood glucose levels [26]. We showed that STZ promoted a hyperglycemic state (Fig 1A and 1E) that mimics a T1DM phenotype [21, 27]. Gliflozins are potent sodium-glucose co-transport 2 inhibitor, with confirmed blood glucose-lowering effects in human patients and mouse models of diabetes [28–30]. Williams et. al demonstrated that empagliflozin reduced blood glucose levels in rats with superimposition of type 1 diabetes and hypertension [31]. In our study, CANA reversed blood glucose levels in both male and female LinSTZ mice (Fig 1A and 1E), corroborating findings in the literature.

Hyperglycemia impairs water reabsorption, leading to dehydration. LinSTZ mice had increased water intake compared to control Lin animals (Fig 1C and 1G), while urinating a volume equivalent to their body weight per day (Fig 1D and 1H), as observed previously observed by our research group [24, 32]. CANA was able to reverse polydipsia and polyuria in male LinSTZ mice (Fig 1C and 1D), but not in females (Fig 1G and 1H). Although we have not monitored ketone production in our model, our first hypothesis would be that the persistence of water intake and elevated urination could be related to ketoacidosis. Such a condition is more commonly present in untreated type 1 diabetes, yet in rare cases, it can observed in patients receiving SGLT2 inhibitors [33–35]. An alternative explanation could be the presence of nephrogenic diabetes insipidus, where the connecting tubules and collecting ducts become less sensitive to vasopressin, impairing the ability to concentrate urine, leading to persistent polydipsia and polyuria independent of glycemia level [36].

Tubuloglomerular feedback (TGF) is directly affected by elevated blood glucose levels. In hyperglycemic conditions, the hyperactivation of the SGLT2 leads to an increase in both glucose and sodium reabsorption and impairs the TGF system. The reduction in Na^+^ delivery in the macula densa promotes dysregulation of TGF, thus increasing renal perfusion by inadequate arterial hemodynamic tone and glomerular hyperfiltration. Inhibition of the SGLT-2 cotransporter increases Na^+^ delivery to the macula densa, restores TGF, and reduces glomerular hypertension and normal glomerular filtration. The estimated glomerular filtration (eGFR) rate was not statistically significantly different in LinSTZ compared to Lin (Table 1). While males LinSTZ showed a trend to normalize the GFR, females displayed elevated eGFR (Table 1). A recent longitudinal study has shown that glomerular hyperfiltration predicts impairment in kidney function and risk of mortality [37]. Thus, CANA ability to reduce hyperfiltration could be beneficial at long-term.

Creatinine clearance, urine chloride, potassium, and urea nitrogen were normalized by CANA treatment in males (Table 1). Yet, female LinSTZ + CANA only presented an amelioration of chloride levels. As mentioned before, female LinSTZ + CANA had persistent elevated water intake and urine production. Therefore, it indicates that inhibition of SGTL2 was enough to partially improve glomerular hyperfiltration and restore ion homeostasis in males, but not in females.

Previous studies from our research center have shown that LinSTZ mice develop accelerated nephropathy [22, 24]. It is also known that patients presenting concomitant diabetes and hypertension have increased chances to develop diabetic nephropathy and chronic kidney disease [38, 39]. Here we show that male LinSTZ mice have increased albuminuria (Fig 2A), injury score (Fig 2D and 2F), and fibrosis (Fig 2E and 2F). CANA treatment partially decreased albuminuria and injury scores in males. Models of type 2 diabetes using male mice have shown canagliflozin and other SGLT2 inhibitors promote protection against albuminuria, kidney injury, and decline in kidney function [40–42]. Conversely, diabetic mice (STZ) lacking SGLT2 still display kidney fibrosis, potentially due to elevated glucose levels in other tubular segments [6].

Female LinSTZ also displayed kidney injury and fibrosis (Fig 3D–3F), and increased ACR at 8w post-STZ. At endpoint, both groups LinSTZ and LinSTZ + CANA had lower levels of ACR compared to 8w post-STZ injections. The lowered ACR in CANA treated female mice is potentially an adaptative mechanism, rather than a direct protective of the drug. No protection against kidney injury or fibrosis was observed. Right and left kidney weight/tibia length ratios were increased in females LinSTZ + CANA (Fig 3B and 3C), a classic hypertrophic response associated with hyperfiltration [43]. Sex-dependent effects of canagliflozin were not found in clinical studies [13, 15, 44]. By performing a systematic review on SGLT2 inhibitors in diabetic nephropathy in rodent models, Ashfaq et. al have shown that only four studies, out of 105, included females; the limited number of studies that did showed that SGLT2 inhibitors lacked effect more often than not [16]. To our knowledge, there were no studies conducted in females to evaluate the impact of CANA in the kidney of diabetic mice. Further investigation is necessary to understand the sex differences in kidney-related alterations by CANA or other SGLT2 inhibitors.

Hyperglycemia is associated with dysregulated lipid metabolism and liver dysfunction [24, 45, 46]. In our study, we observed an increase in triglyceride (TG) levels in males LinSTZ, while CANA mice had lower levels TG compared to LinSTZ (Fig 4B). Impaired insulin secretion or insulin resistance interferes with lipolysis, augmenting circulating fatty acids [47]. Accumulation of fatty acids can disturb the mobilization and β-oxidation in the liver, a risk factor for liver dysfunction. The use of CANA in clinics shows promising results against liver disease, by normalizing circulating levels of liver enzymes and lipid metabolism [25, 48]. CANA is known to promote a global metabolic switch toward lipid utilization [49], thus recovering liver β-oxidation and protecting against liver dysfunction. In our model, CANA treatment in male was enough to normalize ALT levels (Fig 4D). Nonetheless, LinSTZ females treated with CANA had elevated levels of AST and ALT (Fig 3G and 3H), another indicative of differential mechanism of action in our disease model.

Considering the findings in numerous clinical trials utilizing SGLT2 inhibitors, we did not expect to find sex-specific effects with CANA treatment. Still, we observed striking sex-differences in kidney morphology and physiology. In hyperglycemia, excess glucose is reabsorbed by PTCs in an ATP-dependent manner. Such ATP demand is fulfilled by augmented mitochondrial oxidative phosphorylation. Due to high oxygen consumption rate, PTCs become hypoxic, triggering inflammation, increasing production of reactive oxygen species (ROS), cell death, and fibrosis. Although mechanisms of kidney protection by SGLT2 inhibition are still incompletely understood, decreasing glucose uptake by PTCs can decrease the detrimental effects related by SGLT2 hyperactivity. Although SGLT2i efficacy in reducing blood glucose levels was the same in males and females, we hypothesize that kidney-specific effects of CANA in our models differs between sexes.

We acknowledge that our study was limited to a fixed timepoint of intervention, as well duration of CANA treatment. We could not extrapolate our findings to the long-term effects of CANA in the chosen model. It is important to note that our original study design did not consider finding sex-differences with CANA, which imposes a limitation by an unequal statistical power within sexes [50]. Moreover, reporting the data obtained with males and females separately could potentially introduce a bias of “hypothesizing after results are known” [51]. Additionally, females are more resistant to STZ-induced diabetes type 1, thus we must take into consideration the different stages of the disease progression in males and females. Studies have shown that females treated with the same dose of STZ have lower blood glucose levels compared to males [52] but females develop a more severe diastolic dysfunction, despite lower blood glucose levels [53].

In summary, our data provides evidence of the efficacy of canagliflozin in kidney protection against alterations induced by concomitant hypertension and diabetes type 1 in males. Further investigation is necessary to understand the mechanisms behind the lack of protection in females, taking in consideration sexual dimorphisms.

## Supporting information

S1 Data (XLSX)
